# Fish consumption and risk of prostate cancer or its mortality: an updated systematic review and dose–response meta-analysis of prospective cohort studies

**DOI:** 10.3389/fnut.2023.1221029

**Published:** 2023-08-01

**Authors:** Niloofar Eshaghian, Neda Heidarzadeh-Esfahani, Hakimeh Akbari, Gholamreza Askari, Omid Sadeghi

**Affiliations:** ^1^Student Research Committee, Isfahan University of Medical Sciences, Isfahan, Iran; ^2^Department of Nutritional Science, School of Nutritional Science and Food Technology, Kermanshah University of Medical Sciences, Kermanshah, Iran; ^3^Cellular and Molecular Research Center, Gerash University of Medical Sciences, Gerash, Iran; ^4^Nutrition and Food Security Research Center and Department of Community Nutrition, School of Nutrition and Food Science, Isfahan University of Medical Sciences, Isfahan, Iran

**Keywords:** fish, prostate cancer, mortality, dose–response, meta-analysis

## Abstract

Since the release of the last meta-analysis on the association between fish intake and prostate cancer risk, several cohort studies have been published. Moreover, none of the previous meta-analyzes examined the dose–response association between fish intake and prostate cancer. Therefore, the current dose–response meta-analysis was conducted to summarize available findings on the associations of fish intake with the risk of prostate cancer in men. Online databases of PubMed, Scopus, and Web of Science were systematically searched up to September 2022. We included prospective cohort studies that examined the associations of fish intake with the risk of prostate cancer (total, localized, and advanced prostate cancer), its mortality, and cancer progression. Summary relative risks (RR) and 95% confidence intervals (CI) were calculated for the highest versus lowest categories of fish intake using random-effects models. Also, linear and non-linear dose–response analyzes were conducted. In total, 25 prospective cohort studies, recruiting 1,216,474 men, were included in the systematic review, and 22 studies were included in the meta-analysis. During the follow-up periods, ranging from 6 to 33 years, a total of 44,722 cases of prostate cancer were recorded. The comparison between the highest and lowest intakes of total fish revealed the summary RRs of 0.97 (95% CI: 0.86–1.10) for total, 1.01 (95% CI: 0.91–1.13) for advanced, and 0.90 (95% CI: 0.72–1.12) for localized prostate cancer, indicating no significant association. Moreover, the summary RR was 0.55 (95% CI: 0.33–0.92) for prostate cancer mortality and 0.84 (95% CI: 0.65–1.10) for prostate cancer progression, indicating an inverse association between fish intake and prostate cancer mortality. Also, in the dose–response analyzes, each 20 gram/day increase in total fish intake was associated with a 12% lower risk of prostate cancer mortality. Our findings support the protective association between total fish intake and the risk of prostate cancer mortality.

## Introduction

Prostate cancer is the most prevalent cancer in men and the fifth cause of cancer-related death among this population (1). Based on global estimates in 2020, approximately 1.4 million new cases of prostate cancer were recorded, of them, 375,000 patients died (1). In addition, prostate cancer imposes a high economic burden on affected patients and the health care system (2). Despite the high prevalence of prostate cancer, little evidence is available about its etiology and appropriate strategies to control this cancer (3).

Several risk factors including age, ethnicity (African and black ethnic), family history, genetics, smoking, alcohol consumption, and obesity have been identified for prostate cancer (1). Among modifiable risk factors, diet plays an important role (4, 5). It has been shown that adherence to healthy diets such as the Mediterranean diet is associated with a reduced risk of prostate cancer (6, 7). However, it is not clear which components of the Mediterranean diet are involved in this beneficial effect. Recently, some studies have shown that the consumption of fish can affect the prostate cancer risk (8–32). Fish contains a high amount of long-chain omega-3 fatty acids that induce anti-inflammatory effects (33). Inflammation is a potential risk factor for some cancers (34–36). In contrast, some reports are available on the increased risk of certain cancers after intake of omega-3 fatty acids and their dietary sources (37, 38). In addition to this inconsistency, findings from observational studies on the relationship between fish consumption and prostate cancer risk are conflicting. Some studies reported that fish consumption was associated with a reduced risk of prostate cancer (27, 30, 39), while others did not find any significant association (9, 10, 18, 20).

Two meta-analyzes (40, 41) summarized available findings on the link between fish intake and the risk of prostate cancer. However, since the release of the last meta-analysis, several studies have assessed this association (16, 29, 30). In addition, Dai et al. included an effect size from a review article that the effect size was related to Allen et al. (8) study which was also included in the Dai et al. meta-analysis (duplicate effect size) (42). Also, previous meta-analyzes focused on the comparison of prostate cancer risk between the highest and lowest intakes of fish and none of them examined the dose–response association between fish intake and prostate cancer risk. Since the highest and lowest intakes of fish are different among different countries, a meta-analysis by only comparing the intakes might present misleading results. The dose–response meta-analysis can handle this problem by considering the risk of prostate cancer in different categories of fish intakes. Therefore, we conducted this study to summarize available findings on the association between fish intake and prostate cancer risk by performing an updated systematic review and dose–response meta-analysis on prospective cohort studies.

## Methods

The current systematic review and meta-analysis were conducted based on the Preferred Reporting Items for Systematic Reviews and Meta-Analyzes (PRISMA) (43). This meta-analysis was registered at the PROSPERO with code CRD42022347784 (available in Supplementary Files).

### Search strategy

We performed a comprehensive literature search in the online databases of PubMed, Scopus, and Web of Science up to September 12, 2022, to identify prospective cohort studies that examined the association between fish consumption and prostate cancer risk. Supplementary Table S1 shows medical subject heading terms (MESH) and non-MESH terms used in the search strategy. In addition to keywords related to exposure and outcome, we used some terms related to study design (observational OR prospective OR cohort OR hazard OR longitudinal OR historical) to increase the accuracy of search results. This approach was also used in previous meta-analyzes (44, 45). No restriction was considered on language and time of publications. All results in the systematic search were included in the Endnote software. Then, duplicate citations were removed. Study selection was conducted by two reviewers (NE and NHE). After the selection of eligible papers, the reference list of those papers and relevant reviews were screened to avoid missing any publications. We also performed a web-based search in Google Scholar to find any missing articles (Supplementary Table S1). In this search engine, we first searched the combination of “fish” and “prostate cancer” keywords and then we sorted the results by relevance to the searched keywords (not time of publication) and finally screened the first 500 papers.

### Inclusion criteria

We included prospective cohort studies that considered the intakes of fish or its products as an exposure variable and the risk of prostate cancer (including total, localized, and advanced prostate cancer), its mortality, and cancer progression as an outcome variable. All types of fish and its products (including white fish, fatty fish, lean fish, canned tuna, dark meat fish, shrimp, scallops, shellfish, salted fish, smoked fish, broiled fish, fried fish, etc.) were considered in the search strategy and study selection. Other inclusion criteria were reporting relative risk estimates including odds ratio (OR), risk ratio (RR), hazard ratio (HR), incidence rate ratio (IRR), or mortality rate ratio (MRR) along with 95% confidence interval (CI) for the association between fish consumption and risk of prostate cancer or presenting required data for the calculation of these effect sizes. If findings from one dataset were published in >1 article, we selected the most recent version meaning the one with the greatest number of cases or longer follow-up period.

### Exclusion criteria

In the current meta-analysis, we excluded letters, comments, short communications, reviews, meta-analyzes, cross-sectional, case–control, ecological and animal studies, book chapters, and those studies with insufficient data. Moreover, studies that assessed a dietary pattern containing a high amount of fish in relation to prostate cancer were excluded. Studies that assessed the combined effects of fish and other food groups with prostate cancer were excluded as well.

### Data extraction

Required data were extracted from each eligible study by two independent researchers (NE and OS), and any disagreement between them was resolved by discussion with a third researcher. We designed an Excel-based form for data extraction, in which the following information, from each eligible study, was imported to it: the name of the first author, year of publication, cohort name, study location, age range, or the average age of participants, number of participants and cases of prostate cancer, duration of follow-up, methods used to assess dietary intakes and prostate cancer diagnosis, relative risk estimates reported for the link between fish consumption and prostate cancer, and confounding variables adjusted in the statistical analysis. If a study reported stratified relative risks based on a specific variable, we first combined the relative risks using a fixed-effects model, and then, the combined relative risk was included in the main analysis (46).

### Quality assessment

We used the Newcastle-Ottawa scale (NOS), designed for prospective studies, to determine the quality of the studies included in the current meta-analysis (47). Based on this scale, a maximum of 9 points would be assigned to each study according to the following parameters: 4 points for the selection of participants, 2 points for comparison, and 3 points for the length of follow-up and the evaluation of outcome. Since the median score of included studies was 7 in the current meta-analysis, the studies with a score of ≥7 were considered high-quality studies.

### Statistical analysis

All studies presented effect sizes required for the meta-analysis and therefore we conducted no calculation for obtaining the required effect sizes. We included the relative risk estimates (RR, HR, IRR, MRR) of prostate cancer, reported for the comparison between the highest and lowest intakes of fish, in the meta-analysis. None of the included studies reported OR for the association between fish intake and prostate cancer risk. In addition, since the prevalence of prostate cancer was rare (<10%) in the population of cohorts included in the current meta-analysis, we did not convert the HRs to RRs. To calculate the summary relative risk, a random-effects model was used to take between-study heterogeneity into account. Also, we used both Q-statistic and I^2^ values to assess heterogeneity among the included studies. *I*^2^ values greater than 50% were considered significant between-study heterogeneity (48). In case of significant between-study heterogeneity, we conducted subgroup and meta-regression analyzes based on study location [United States (US) vs. non-US countries], duration of follow-up (≥10 vs. < 10 years), sample size (≥ 10,000 vs. < 10,000 participants), adjustment for energy intake and body mass index (BMI) (adjusted vs. unadjusted), and study quality (≥ 7 vs. < 7 scores) to detect possible sources of heterogeneity. We also assess publication bias using Egger’s regression asymmetry test for the associations with ≥10 observations (49). To detect the effect of probable missing studies on the overall relative risk, the trim-and-fill method was used (50). In addition, sensitivity analysis using a random-effects model was performed to examine the influence of each study on the overall estimates. This analysis repeated the main analysis by removing one study each time.

To assess the linear association between fish intake and prostate cancer risk, we used the one-stage dose–response meta-analysis (51). In this method, we need the total number of participants and prostate cancer cases and the relative risks of prostate cancer in each category of fish intake. To link the dosage of fish intake with the relative risks, we assigned the median or mean amount of fish intake in each category of fish intake to the corresponding relative risk in each study. This was done for studies that reported the mean or median intakes of fish in each category. For studies that reported the intakes as ranges, we estimated the midpoint in each category (52, 53). When the highest category was open-ended, the length of the open-ended interval was assumed to be the same as that of the adjacent interval (52, 53). For studies that reported the intakes of fish as time per week/month, we converted them to gram/day by considering the standard portion of 150 gram for a single fish meal (28). The possible non-linear dose–response associations were examined using restricted cubic splines with 3 knots at percentiles of 10, 50, and 90% of the distribution (54). The correlation within each set of provided relative risks was taken into account and the study-specific estimates were combined by using a one-stage linear mixed-effects meta-analysis (51). This method estimates the study-specific slopes and combines them to obtain an overall average slope in a single stage (51). The significance for non-linearity was calculated by null hypothesis testing, in which the coefficient of the second spline was considered equal to zero (51). Statistical analyzes were conducted using STATA version 14.0. *p* < 0.05 was considered statistically significant for all tests except for Cochran’s Q test which was considered significant at *p* < 0.10 (55).

## Results

### Literature search

**Figure 1:**
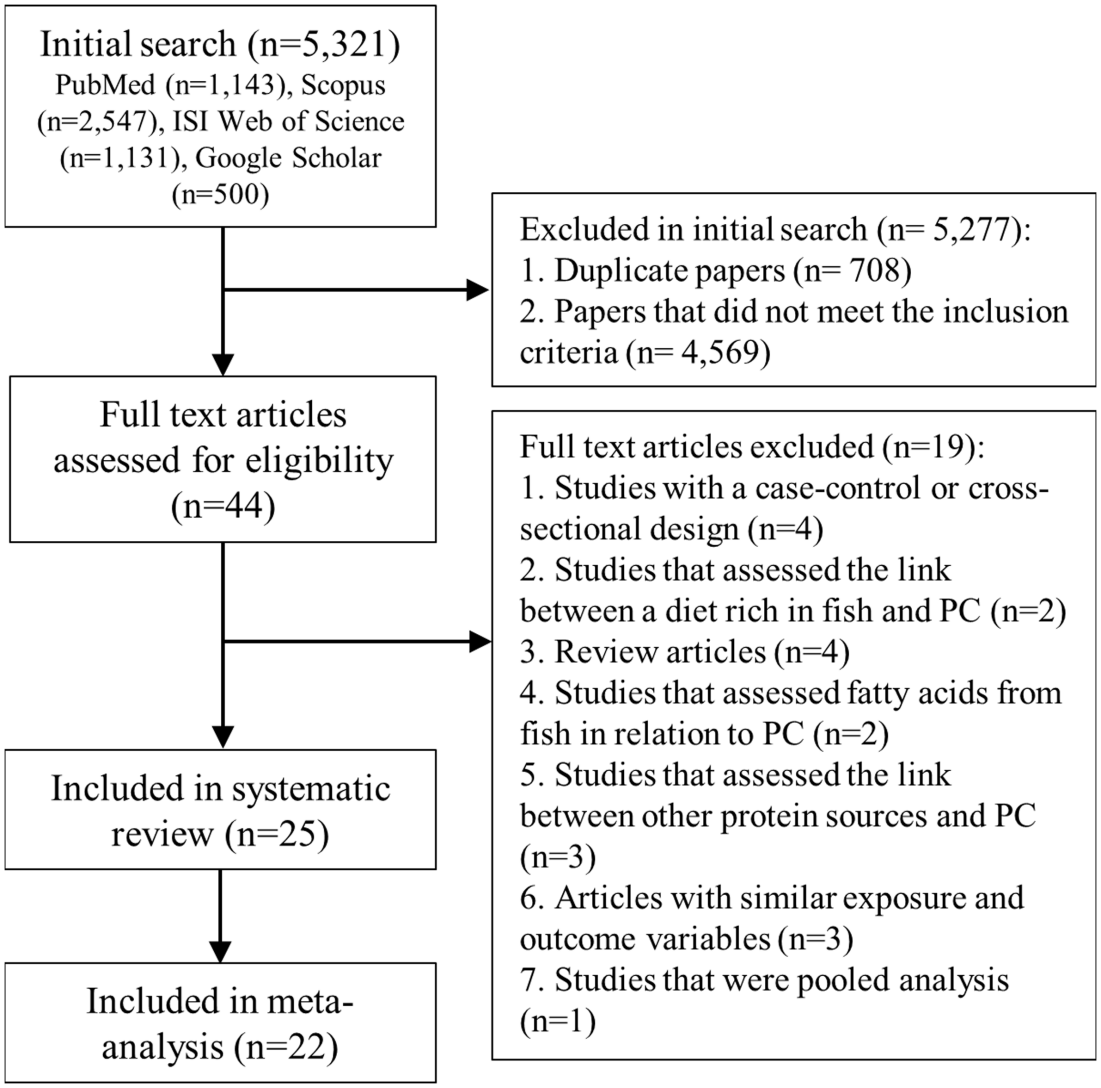
Flow diagram of study selection. PC, prostate cancer.

We identified 5,321 articles in our initial search. After the exclusion of duplicate papers and those that did not meet the inclusion criteria, 44 full-text articles of potentially relevant studies were identified. After the full-text review, we excluded an additional 4 studies because they had a case–control or cross-sectional design (56–59). Also, two studies assessed the link between a diet rich in fish and prostate cancer, and therefore, were excluded (6, 60). Four articles were excluded because they were review articles (61–64). Two studies that assessed fatty acids from fish in relation to prostate cancer (65, 66) and three articles that evaluated intakes of poultry or other protein sources rather than fish intake were excluded as well (67–69). We found 6 articles published on the datasets of Physician’s Health Study (PHS) (13, 15), Health Professionals Follow-up Study (HPFS) (10, 12, 15), and National Institutes of Health-American Association of Retired Persons (NIH-AARP) Diet and Health Study (11, 16). However, since these studies assessed different types of exposure (fish or fish products) and outcome variables, we included them in this meta-analysis. Also, we found two duplicate papers on the European Prospective Investigation into Cancer and Nutrition (EPIC) study (9, 70), in which the one with the greatest number of prostate cancer cases was included (9), and the other one was excluded (70). In addition, two duplicate papers were found on the PHS (13, 71) and two on the Netherlands Cohort Study (NLCS) (25, 72), of them, the one with a greater number of cases was included (13, 25) and the duplicate papers were excluded (71, 72). Furthermore, we found a pooled analysis of 15 prospective cohort studies that included most studies evaluated in the current meta-analysis. Therefore, that pooled analysis was excluded from the current meta-analysis (73). After the above-mentioned exclusions, 25 articles from prospective cohort studies were included in the systematic review (8–32) and 22 articles were included in the meta-analysis (8–14, 17–27, 29–32). Among them, 17 articles reported risk estimates for prostate cancer (8–10, 13, 16–20, 23–28, 30, 32), 8 articles for advanced prostate cancer (10, 16, 19, 20, 23, 25, 28, 32), 9 publications for prostate cancer mortality (11, 13–16, 19, 21, 27, 29), 3 articles for prostate cancer progression (12, 22, 31), and 3 articles for localized prostate cancer (23, 25, 28). Three studies reported risk estimates for fatal prostate cancer, in which all fatal cases were dead during the follow-up periods. Therefore, in the current meta-analysis, fatal prostate cancer was considered similar to prostate cancer mortality. Also, included articles assessed intakes of total fish (8–14, 17–27, 29–32), fatty fish (9, 15, 19), lean fish (19), white fish (9), dark meat fish (13), shrimp, lobster, or scallops (13), canned tuna (13, 16), salted or smoked fish (28), fried fish (31), and shellfish (20) in relation to prostate cancer. Figure 1 shows the flow diagram of the study selection.

### Characteristics of included studies

The characteristics of included studies are provided in Table 1. The total number of participants in these studies ranged from 940 to 293,464 people, with an age range between 25 and 96 years. In total, 1,216,474 participants were enrolled in the 25 publications included in the current systematic review. During the follow-up periods, ranging from 6 to 33 years, a total of 44,722 cases of prostate cancer, 5,422 cases of advanced prostate cancer, 3,779 deaths from prostate cancer, 613 cases of prostate cancer progression, and 406 cases of localized prostate cancer were recorded. Out of 25 articles, 15 articles were from the US (10–18, 20, 22, 23, 26, 29, 31), 7 articles from Europe (9, 19, 25, 27, 28, 30, 32), and 3 papers from Asia (8, 21, 24). Dietary intake of fish was assessed using a validated food frequency questionnaire (FFQ) in 18 articles (8, 10–13, 15, 16, 18–20, 22–25, 28, 29, 31, 32), a non-validated questionnaire in 5 publications (14, 17, 21, 27, 30), both validated FFQ and diet history in 1 article (9), and both validated FFQ and dietary recall in 1 article (26). Among the included studies, intakes of total fish, white fish, fatty fish, lean fish, canned tuna, dark meat fish, shrimp, scallops, shellfish, salted fish, smoked fish, broiled fish, and fried fish were assessed with prostate cancer risk. However, the number of studies for total fish was sufficient for performing a meta-analysis. Prostate cancer was assessed using medical records or cancer registries in 20 studies (8, 11, 12, 14, 16, 17, 19–32), self-reported data in 3 studies (10, 15, 18), and both medical records and self-reported data in 2 studies (9, 13). In most articles, relative risks were adjusted for age (*n* = 21), BMI (*n* = 12), smoking (*n* = 12), alcohol consumption (*n* = 7), physical activity (*n* = 11), energy intake (*n* = 12), other dietary variables (*n* = 15), and family history of prostate cancer (*n* = 6). The NOS scores of the included studies were between 5 and 8. Looking at the variation of NOS scores and considering the score of 7 as a median, 17 articles had a score of ≥7, defined as high-quality studies (8–12, 14, 16, 17, 19, 20, 22–24, 26, 29, 30, 32) (Supplementary Table S2).

**Table 1:** Characteristics of included studies on the associations between fish intake and risk of prostate cancer in men aged ≥18 years^1^.

Author	Country/study name	Age (*y*^2^)	Sample size (*n*)	Follow up (*y*^3^)	Case (*n*)	Exposure	Exposure assessment	Outcome	Outcome assessment	(Median/ cutoff point)	ES (95% CI)	Adjustment
Allen et al. (8)	Japan/LSSC	40–75	18,115	33	196	Fish	FFQ: self-reported	PC	Medical records	<2 time/wk. 2–4 time/wk. Daily	1.00 RR: 1.18 (0.83–1.67) RR: 1.54 (1.03–2.31)	Age, calendar period, city of residence, radiation dose, education
				31	153	Broiled fish				<2 time/wk. 2–4 time/wk. Daily	1.00 RR: 1.22 (0.87–1.72) RR: 1.25 (0.48–3.28)	
				17	153	Total fish				Low Intermediate High	1.00 RR: 1.19 (0.82–1.73) RR: 1.77 (1.01–3.11)	
Allen et al. (9)	Europe/EPIC	33–67	142,520	15	2,727	Total fish	FFQ/diet history: self-reported/ interview	PC	Medical records/ self-reported	13 g/d 13 g/d 17 g/d 25 g/d 43 g/d	1.00 HR: 1.00 (0.87–1.14) HR: 1.09 (0.96–1.25) HR: 1.02 (0.90–1.17) HR: 1.05 (0.91–1.20)	Education, marital status, height, weight, intake of energy
					2,021	White fish				9 g/d 13 g/d 16 g/d 20 g/d 32 g/d	1.00 HR: 1.08 (0.91–1.27) HR: 0.97 (0.83–1.13) HR: 0.99 (0.86–1.14) HR: 1.03 (0.90–1.18)	
					2,727	Fatty fish				34 g/d 90 g/d 200 g/d 265 g/d 466 g/d	1.00 HR: 1.04 (0.91–1.19) HR: 1.05 (0.94–1.19) HR: 1.03 (0.92–1.17) HR: 1.07 (0.95–1.21)	
Augustsson et al. (10)	US/HPFS	40–75	47,882	12	2,482	Total fish	FFQ: self-reported	PC	Self-reported	4.30 g/d 15.7 g/d 53.5 g/d 75.0 g/d	1.00 RR: 1.05 (0.91–1.21) RR: 1.06 (0.93–1.20) RR: 0.93 (0.80–1.08)	Age, dietary intakes of energy, fatty acids, lycopene, retinol, and vitamin D, physical activity
					617			Advanced PC		4.30 g/d 15.7 g/d 53.5 g/d 75.0 g/d	1.00 RR: 1.05 (0.79–1.39) RR: 1.14 (0.88–1.46) RR: 0.83 (0.61–1.13)	
					278			Metastatic PC		4.30 g/d 15.7 g/d 53.5 g/d 75.0 g/d	1.00 RR: 0.71 (0.48–1.06) RR: 0.82 (0.58–1.16) RR: 0.56 (0.37–0.86)	
Bosire et al. (11)	US/NIH-AARP	50–71	293,464	11	428	Total fish	FFQ: self -reported	Fatal PC	Medical records	49.5 g/d 148 g/d	1.00 HR: 0.79 (0.65–0.96)	Age, race, education, BMI, smoking, physical activity, family history of PC, diabetes, intake of energy, history of PSA screening, and all other components in the specific index
Chan et al. (12)	US/HPFS	40–75	1,202	14	392	Total fish	FFQ: self -reported	PC progression	Medical records	Q1 Q2 Q3 Q4	1.00 HR: 1.01 (0.76–1.35) HR: 0.94 (0.69–1.29) HR: 0.73 (0.52–1.02)	Age, energy intake, pre-diagnostic diet, all other post-diagnostic food groups
Chavarro et al. (13)	US/PHS	40–84	20,167	23	2,161	Total fish	FFQ: self -reported	PC	Self-reported	15 g/d 33 g/d 61.5 g/d 145.5 g/d	1.00 RR: 1.04 (0.91–1.18) RR: 0.98 (0.87–1.10) RR: 1.11 (0.95–1.30)	Age, BMI, physical activity, intake of alcohol, tomato, dairy products, smoking, race, use of multivitamins, vitamin E supplements, random assignment to aspirin or beta-carotene, tumor stage and grade at diagnosis, clinical presentation of case
						Canned tuna				< 1 time/mo 1–3 time/mo 1 time/wk. ≥ 2 time/wk	1.00 RR: 1.01 (0.91–1.12) RR: 1.00 (0.89–1.13) RR: 1.12 (0.95–1.31)	
						Dark meat fish				< 1 time/mo 1–3 time/mo ≥ 1 time/wk	1.00 RR: 0.99 (0.91–1.09) RR: 0.91 (0.79–1.05)	
						Other fish				< 1 time/mo 1–3 time/mo 1 time/wk. ≥ 2 time/wk	1.00 RR: 1.14 (0.99–1.31) RR: 1.09 (0.94–1.26) RR: 1.33 (1.13–1.58)	
						Shrimp, lobster, scallops				< 1 time/mo 1–3 time/mo ≥ 1 time/wk	1.00 RR: 1.00 (0.91–1.09) RR: 0.93 (0.80–1.08)	
			2,161		230	Total fish		PC mortality	Medical records	15 g/d 33 g/d 61.5 g/d 145.5 g/d	1.00 RR: 0.73 (0.50–1.08) RR: 0.76 (0.54–1.08) RR: 0.52 (0.30–0.91)	
						Canned tuna				< 1 time/mo 1–3 time/mo 1 time/wk. ≥ 2 time/wk	1.00 RR: 0.65 (0.47–0.89) RR: 0.85 (0.58–1.25) RR: 0.57 (0.33–0.99)	
						Dark meat fish				< 1 time/mo 1–3 time/mo ≥ 1 time/wk	1.00 RR: 0.76 (0.57–1.02) RR: 0.64 (0.39–1.04)	
						Other fish				< 1 time/mo 1–3 time/mo 1 time/wk. ≥ 2 time/wk	1.00 RR: 0.66 (0.45–0.98) RR: 0.70 (0.47–1.03) RR: 0.58 (0.35–0.97)	
						Shrimp, lobster, scallops				< 1 time/mo 1–3 time/mo ≥ 1 time/wk	1.00 RR: 0.85 (0.63–1.15) RR: 1.10 (0.72–1.67)	
Hsing et al. (14)	US/LBC	≥35	17,633	20	149	Total fish	Researcher-made questionnaire: self-reported	Fatal PC	Medical records	4 g/d 6 g/d 14.5 g/d 25.5 g/d	1.00 RR: 1.10 (0.70–1.60) RR: 0.90 (0.60–1.40) RR: 0.80 (0.50–1.30)	Age, tobacco use
Kenfield et al. (15)	US/HPFS	40–75	42,701	20	576	Fatty fish	FFQ: self-reported	Lethal PC	Self-reported	1 serv/wk. ≥1 serv/wk	1.00 HR: 0.83 (0.64–1.07)	Age, race, diabetes, use of multivitamin, vitamin E, random assignment status
	US/PHS	40–84	20,324	23	337					1 serv/wk. ≥1 serv/wk	1.00 HR: 0.80 (0.57–1.14)	
Lan et al. (16)	US/NIH-AARP	50–71	159,482	14	17,349	Canned tuna	FFQ: self-reported	PC	Medical records	≤11 time/y 3 time/mo 1–2 time/wk. ≥3 time/wk	1.00 HR: 1.02 (0.98–1.06) HR: 1.03 (0.99–1.08) HR: 0.95 (0.87–1.03)	Age, intake of energy, race, family history of PC, education, marital status, smoking, waist circumference, BMI, physical activity, PSA and digital rectal examination screening history, diabetes, father’s occupation, height, intake of grains, vegetables, fruits, potatoes, dairy products and sweets, animal products, alcohol
					2,297			Advanced PC		≤11 time/y 3 time/mo 1–2 time/wk. ≥3 time/wk	1.00 HR: 0.97 (0.87–1.07) HR: 1.08 (0.96–1.21) HR: 1.01 (0.81–1.25)	
					804			Fatal PC		≤11 time/y 3 time/mo 1–2 time/wk. ≥3 time/wk	1.00 HR: 0.94 (0.79–1.12) HR: 1.00 (0.81–1.23) HR: 1.03 (0.71–1.49)	
Le Marchand et al. (17)	US/NR	NR	20,316	14	198	Total fish	Researcher-made questionnaire: interviews	PC	Medical records	7 g/d 19 g/d 31 g/d 43 g/d	1.00 RR: 1.10 (0.70–1.70) RR: 0.90 (0.60–1.30) RR: 1.20 (0.80–1.80)	Age, race, income
Mills et al. (18)	US/AHS	≥25	14,000	6	180	Total fish	FFQ: self-reported	PC	Self-reported	0 g/d 10.7 g/d 32.1 g/d	1.00 RR:1.37 (0.95–1.96) RR: 1.57 (0.88–2.78)	Age, education, intake of meat, poultry, beans, legumes or peas, citrus fruit, dry fruit, nuts, tomatoes, index of fruit
Outzen et al. (19)	Denmark/ DDCHC	50–64	26,749	19	1,886	Total fish	FFQ: self -reported	PC	Medical records	20.85 g/d 34.75 g/d 50.90 g/d 69.30 g/d	1.00 IRR: 1.14(0.99–1.30) IRR: 1.05 (0.91–1.21) IRR: 1.12 (0.97–1.29)	BMI, education, smoking, participation in sport, intake of red and processed meat, dairy products, alcohol, indicator for alcohol abstinence, indicator variable for fish intake
						Lean fish				12.95 g/d 21.65 g/d 31.85 g/d 43.55 g/d	1.00 IRR: 1.04 (0.90–1.19) IRR: 1.06 (0.92–1.22) IRR: 1.09 (0.94–1.27)	
						Fatty fish				4.70 g/d 10.50 g/d 18.15 g/d 27.65 g/d	1.00 IRR: 1.10 (0.96–1.26) IRR: 1.09 (0.95–1.26) IRR: 0.95 (0.82–1.11)	
				19	498	Total fish		Advanced PC		20.85 g/d 34.75 g/d 50.90 g/d 69.30 g/d	1.00 IRR: 1.15 (0.89–1.47) IRR: 0.98 (0.76–1.28) IRR: 1.05 (0.81–1.36)	
						Lean fish				12.95 g/d 21.65 g/d 31.85 g/d 43.55 g/d	1.00 IRR: 1.04 (0.80–1.34) IRR: 1.15 (0.89–1.48) IRR: 0.99 (0.75–1.31)	
						Fatty fish				4.70 g/d 10.50 g/d 18.15 g/d 27.65 g/d	1.00 IRR: 1.05 (0.81–1.35) IRR: 1.05 (0.81–1.36) IRR: 0.91 (0.69–1.20)	
				20	228	Total fish		PC mortality		21.95 g/d 35.25 g/d 51.55 g/d 70.85 g/d	1.00 MRR:0.56(0.37–0.84) MRR:0.88(0.61–1.25) MRR:0.94(0.66–1.35)	
						Lean fish				13.70 g/d 22.10 g/d 32.15 g/d 43.85 g/d	1.00 MRR:1.03(0.71–1.48) MRR:0.77(0.52–1.14) MRR:0.76(0.50–1.14)	
						Fatty fish				5.30 g/d 10.90 g/d 18.15 g/d 27.05 g/d	1.00 MRR:0.91(0.61–1.35) MRR:1.06(0.72–1.57) MRR:1.29(0.87–1.92)	
Park et al. (20)	US/ MCS	≥45	82,483	8	4,404	Total fish	FFQ: self -report	PC	Medical records	2.76 g/d 8.97 g/d 15.18 g/d 23.23 g/d 4.94 g/d	1.00 RR: 1.09 (0.99–1.20) RR: 1.05 (0.95–1.16) RR: 1.11 (1.00–1.22) RR: 1.04 (0.93–1.15)	Time on study, race, family history of PC, education, BMI, smoking, intake of energy
						Shellfish				0.05 g/1000 kcal 0.46 g/1000 kcal 1.38 g/1000 kcal 2.60 g/1000 kcal 5.10 g/1000 kcal	1.00 RR: 0.93 (0.84–1.02) RR: 0.96 (0.87–1.05) RR: 0.99 (0.90–1.09) RR: 1.00 (0.91–1.10)	
					1,278	Total fish		Advanced PC		2.76 g/d 8.97 g/d 15.18 g/d 23.23 g/d 4.94 g/d	1.00 RR: 1.11 (0.93–1.33) RR: 1.04 (0.87–1.26) RR: 1.15 (0.95–1.39) RR: 1.01 (0.82–1.23)	
						Shellfish				0.05 g/1000 kcal 0.46 g/1000 kcal 1.38 g/1000 kcal 2.60 g/1000 kcal 5.10 g/1000 kcal	1.00 RR: 0.89 (0.75–1.06) RR: 0.92 (0.77–1.10) RR: 1.02 (0.86–1.22) RR: 0.97 (0.80–1.17)	
Pham et al. (21)	Japan/ Miyako Study	30–79	5,589	17	21	Total fish	Researcher- made questionnaire: self-reported	PC mortality	Medical records	10 g/d 96 g/d	1.00 HR: 0.12 (0.05–0.32)	Age, smoking, diabetes, employment status, living with spouse and study area, intake of alcohol, vegetable, fruit, meat
Richman et al. (22)	US/ CaPSURE	65	1,294	13	127	Total fish	FFQ: self-reported	PC progression	Medical records	8.57 g/d 27.85 g/d 40.71 g/d 92.14 g/d	1.00 RR: 0.78 (0.45–1.37) RR: 1.07 (0.65–1.73) RR: 1.13 (0.70–1.84)	Age, intake of energy, time from diagnosis to questionnaire, primary treatment, BMI, physical activity, Gleason sum and PSA
Rohrmann et al. (23)	US/ CLUE II cohort study	≥35	3,892	15	199	Total fish	FFQ: self-reported	PC	Medical records	21.42 g/d 64.20 g/d 128 g/d	1.00 HR: 1.12 (0.75–1.67) HR: 0.86 (0.44–1.67)	Age, BMI, intake of energy, saturated fat, tomato products
					54			Advanced PC		21.42 g/d 64.20 g/d 128 g/d	1.00 HR: 1.05 (0.47–2.38) HR: 0.92 (0.27–3.21)	
					74			Localized PC		21.42 g/d 64.20 g/d 128 g/d	1.00 HR: 0.85 (0.41–1.72) HR: 0.62 (0.19–2.06)	
Sato et al. (24)	Japan/ ONHICS	40–79	24,895	7	95	Total fish	FFQ: self-reported	PC	Medical records	13.1 g/d 39.8 g/d 77.1 g/d 124.5 g/d	1.00 HR: 0.92 (0.48–1.76) HR: 0.73 (0.42–1.28) HR: 0.72 (0.40–1.33)	Age, drinking status, smoking, walking, BMI, marital status, intake of energy, calcium, beef, pork, soybean, tomato, green tea
Schuurman et al. (25)	Netherlands/NLCS	55–69	2,167	6	642	Total fish	FFQ: self-reported	PC	Medical records	0 g/d 5 g/d 14 g/d 32 g/d	1.00 RR: 0.83 (0.62–1.11) RR: 0.95 (0.70–1.29) RR: 1.03 (0.80–1.34)	Age, family history of PC, socioeconomic status
					226			Localized PC		0 g/d 25 g/d	1.00 RR: 0.85 (0.58–1.24)	
					213			Advanced PC		0 g/d 25 g/d	1.00 RR: 1.13 (0.78–1.58)	
Severson et al. (26)	US/NR	≥45	7,999	21	174	Total fish	FFQ/dietary recall: interview	PC	Medical records	21.42 g/d 64.20 g/d 128 g/d	1.00 RR: 0.84 (0.61–1.17) RR: 1.22 (0.74–2.01)	Age
Terry et al. (27)	Swedish/Swedish cohort	55.6	6,272	30	466	Total fish	Researcher-made questionnaire: self-reported	PC	Medical records	Never/seldom Small part Moderate part Large part Never/seldom Small part Moderate part Large part	RR: 2.30 (1.20–4.50) RR: 1.20 (1.00–1.40) 1.00 RR: 1.00 (0.70–1.60)	Age, BMI, physical activity, smoking, intake of alcohol, red meat, processed meat, fruit, vegetables, and milk
					340			PC mortality			RR: 3.30 (1.80–6.00) RR: 1.30 (1.00–1.60) 1.00 RR: 0.90 (0.60–1.70)	
Torfadottir et al. (28)	Iceland/AGES-Reykjavik study	67–96	2,054	7	133	Salted or smoked fish	FFQ: self-reported	PC	Medical records	≤1 times/mo >1 times/mo	1.00 HR: 0.99 (0.69–1.42)	Birth year, age, education, family history of prostate disease, going to a physician regularly, height, BMI, diabetes, intake of total fish, fish oil, salted or smoked fish, milk, rye bread, meat
		67–96			27	Salted or smoked fish		Advanced PC		≤1 times/mo >1 times/mo	1.00 HR: 2.28 (1.04–5.00)	
		67–96			106	Salted or smoked fish		Localized PC		≤1 times/mo >1 times/mo	1.00 HR: 0.63 (0.40–1.00)	
Wang et al. (29)	US/ CPS-II	72.1	9,286	24	666	Total fish	FFQ: self-reported	PC mortality	Medical records	4 g/d 21.4 g/d 34 g/d 64 g/d	1.00 RR: 0.92 (0.73–1.15) RR: 0.92 (0.74–1.15) RR: 0.87 (0.69–1.09)	Age, calendar year of diagnosis, tumor extent, Gleason score, nodal involvement, education, family history of PC, history of PSA testing, BMI, smoking, physical activity, diabetes, intake of energy, fruit, vegetable, history of CVD and other cancer
Watling et al. (30)	UK/ UK biobank	40–70	217,937	13	9,501	Total fish	Researcher-made questionnaire: self-reported	PC	Medical records	Regular meat-eaters Low meat-eaters Fish-eaters Vegetarians	1.00 HR: 1.00 (0.96–1.04) HR: 0.80 (0.65–0.98) HR: 0.70 (0.55–0.90)	Region of recruitment, height, physical activity, TDI, education, employment status, smoking, intake of alcohol, race, diabetes, BMI, marital status, PSA test
Wilson et al. (31)	US / WUGS	61	940	7	94	Total fish	FFQ: self-reported	PC progression	Medical records	5 g/d 13 g/d 25 g/d 50 g/d	1.00 HR: 0.93 (0.50–1.74) HR: 0.94 (0.51–1.72) HR: 0.83 (0.43–1.63)	Age, race, family history of PC, BMI, smoking, physical activity, clinical stage, pathological stage, Gleason sum, PSA, intake of energy, total calcium, cooked tomato products, coffee
						Fried fish				0 g/d 2 g/d 5 g/d 17 g/d	1.00 HR: 1.72 (0.76–3.89) HR: 1.59 (0.76–3.35) HR: 1.49 (0.70–3.21)	
						Not Fried fish				0 g/d 2 g/d 4 g/d 16 g/d	1.00 HR: 0.77 (0.39–1.52) HR: 0.68 (0.35–1.29) HR: 0.78 (0.44–1.40)	
Wright et al. (32)	Finland/ ATBC Study	50–69	27,111	21	1,929	Total fish	FFQ: self-reported	PC	Medical records	Q1 Q2 Q3 Q4	1.00 RR: 0.99 (0.88–1.12) RR: 0.87 (0.76–0.98) RR: 0.90 (0.79–1.02)	Age, smoking, trial intervention assignment, education, intake of energy, dietary fat
					438			Advanced PC		Q1 Q2 Q3 Q4	1.00 RR: 1.12 (0.86–1.47) RR: 1.08 (0.82–1.41) RR: 1.02 (0.78–1.35)	

^1^PC, prostate cancer; ES, effect size; RR, risk ratio; Max, maximum; n, number; CI, confidence interval; FFQ, food frequency questionnaire; BMI, body mass index; US, United States; NR, Not-Reported; wk, week; LSSC, Life Span Study Cohort; EPIC, European Prospective Investigation into Cancer and Nutrition; HPFS, Health Professional Follow-up Study; NIH-AARP, National Institutes of Health-American Association of Retired Persons; PHS, Physicians’ Health Study; LBC, Lutheran Brotherhood Cohort; DDCHC, Danish ‘Diet, Cancer and Health’ cohort; PSA, Prostate-Specific Antigen; CaPSURE, cancer of the prostate strategic urologic research endeavor; AHS, Adventist Health Study; ONHICS, Osaki National Health Insurance Cohort Study; MCS, Multiethnic Cohort study; NLCS, Netherlands Cohort Study; HR, hazard ratio; CVD, Cardiovascular Disease; d, day; y, year; mo, month. AGES-Reykjavik Study, Age, Gene/Environment Susceptibility–Reykjavik Study; CPS-II, Cancer Prevention Study-II; TDI, Townsend Deprivation Index; UK, United Kingdom; WUGS, Washington University Genetics Study; OR, odd ratio; ATBC Study, Alpha-Tocopherol, Beta-Carotene Cancer Prevention Study; Q, quartile; g/d, gram(s) per day; g/wk, gram(s) per week; serv, serving; IRR, incidence rate ratio; MRR, mortality rate ratio. ^2^Presented as mean or range. ^3^Presented as maximum duration of follow-up.

### Findings from the systematic review

Out of 15 articles on the association between total fish intake and prostate cancer risk, 2 articles indicated an inverse association (27, 30), 1 article showed a positive association (8), and others illustrated no significant association. For advanced prostate cancer, none of the articles revealed a significant association between total fish intake and advanced prostate cancer. In the case of prostate cancer mortality, 4 studies showed an inverse association with total fish intake (11, 13, 21, 27). For prostate cancer progression and localized prostate cancer, none of the included studies revealed a significant association with total fish intake. For the different types of fish products, one article showed an inverse association between canned tuna and the risk of prostate cancer mortality (13) and another article showed a positive association in terms of salted/smoked fish and advanced prostate cancer (28). In addition, two articles assessed “other fish,” in which the type of fish was unclear, and reported a significant positive association with the risk of total prostate cancer (8, 13).

### Findings from the meta-analysis

Data on different types of fish, except total fish, were not sufficient for a meta-analysis. Therefore, our meta-analysis was mainly focused on total fish intake and prostate cancer risk. Of 25 articles included in the systematic review, three assessed only fatty fish, canned tuna, and salted or smoked fish intake rather than total fish intake, and therefore, were excluded from the meta-analysis (15, 16, 28). In total, 22 articles with a total sample size of 991,913 were included in the current meta-analysis.

### Total fish intake and risk of prostate cancer

**Figure 2:**
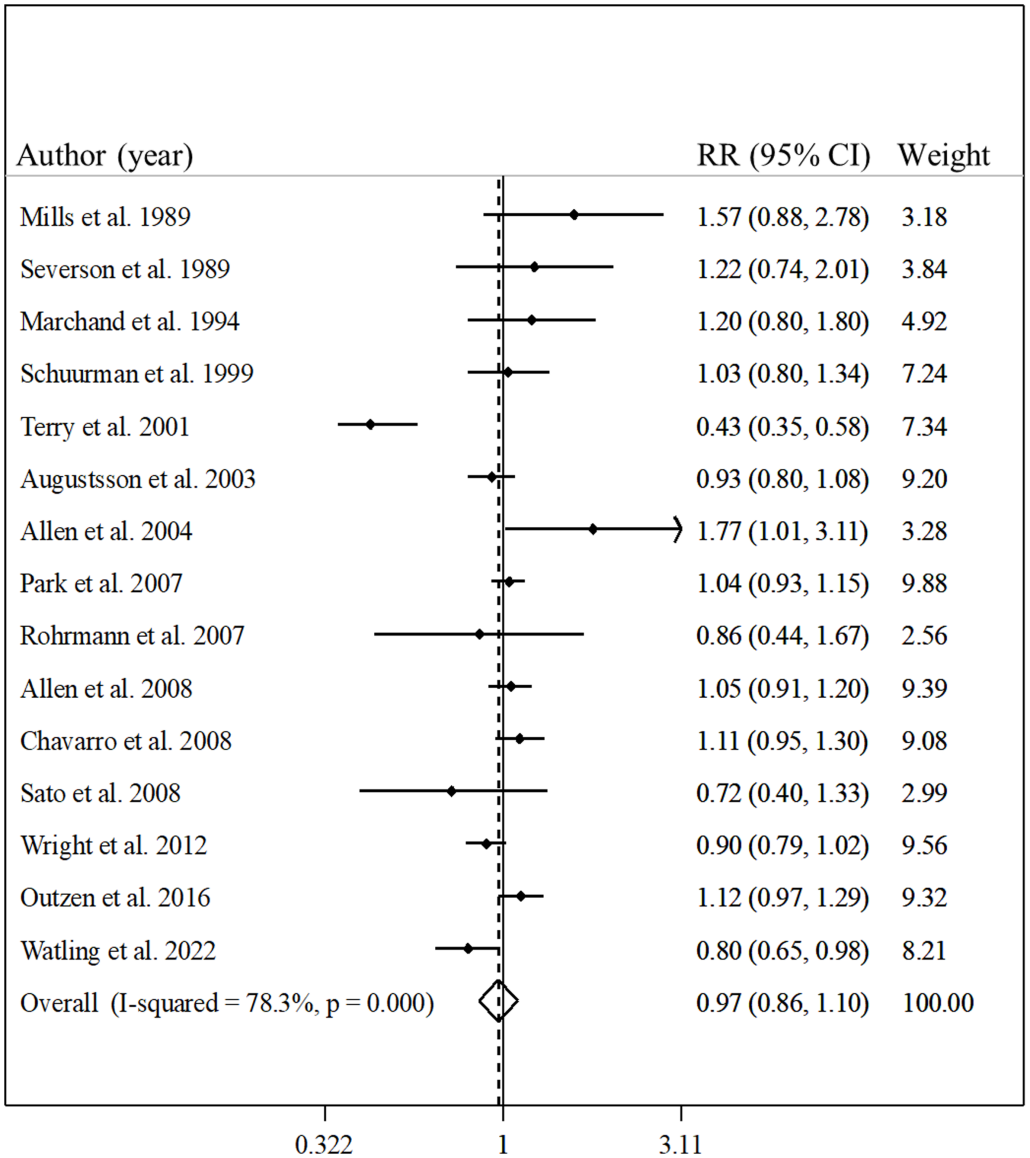
Forest plot for the association between total fish intake and risk of prostate cancer in men aged ≥18 years by comparing the highest and lowest categories of fish intake. The pooled RR was obtained using a random-effects model. RR, relative risk; CI, confidence interval.

Fifteen studies with a total of 662,505 participants and 27,197 prostate cancer cases were included in this association (8–10, 13, 17–20, 23–27, 30, 32). The summary relative risk for the risk of prostate cancer, comparing the highest with the lowest intake of total fish, was 0.97 (95% CI: 0.86–1.10, *p* = 0.63), indicating no significant association between total fish intake and prostate cancer (Figure 2). However, there was evidence of significant heterogeneity among studies (*I*^2^ = 78.3%; *p* < 0.001). In the subgroup analysis, between-study heterogeneity reduced in the subgroups of studies performed in the US, those studies with <10 years’ duration of follow-up, and studies that controlled their analyzes for total energy intake. Findings from the meta-regression revealed that our findings on the association between fish intake and prostate cancer risk were not different in the subgroups of studies (Table 2).

**Table 2:** Subgroup analyzes for the associations between fish intake and risk of prostate cancer in men aged ≥18 years^1^.

	#R*R*^2^	Pooled RR (95% CI)^3^	*I*^2^ (%)^4^	*p*-values	
				heterogeneity^5^	Meta-regression
Total prostate cancer					
Overall	15	0.97 (0.86–1.10)	78.3	<0.001	
Subgroup analysis					
Study location					0.23
US	7	1.04 (0.97–1.12)	0.0	0.43	
Non-US countries	8	0.90 (0.73–1.10)	87.0	<0.001	
Sample size, participants					0.13
<10,000	4	0.81 (0.46–1.42)	89.3	<0.001	
≥10,000	11	1.02 (0.93–1.11)	52.9	0.02	
Follow-up, year					0.64
<10	4	1.04 (0.92–1.18)	12.1	0.33	
≥10	11	0.95 (0.81–1.11)	83.1	<0.001	
Adjustment for energy intake					0.68
No	9	1.02 (0.80–1.29)	86.5	<0.001	
Yes	6	0.98 (0.91–1.05)	9.0	0.36	
Adjustment for BMI					0.16
No	7	1.04 (0.90–1.20)	44.4	0.09	
Yes	8	0.89 (0.73–1.07)	87.0	<0.001	
Study quality^6^					0.47
Low quality	4	0.91 (0.55–1.51)	93.2	<0.001	
High quality	11	1.00 (0.92–1.09)	44.9	0.05	
Advanced Prostate Cancer					
Overall	6	1.01 (0.91–1.13)	0.0	0.84	
Subgroup analysis					
Study location					0.41
US	3	0.95 (0.80–1.13)	0.0	0.58	
Non-US countries	3	1.06 (0.92–1.21)	0.0	0.95	
Sample size, participants					0.54
<10,000	2	1.08 (0.87–1.33)	0.0	0.80	
≥10,000	4	0.99 (0.87–1.12)	0.0	0.67	
Follow-up, year					0.57
<10	2	1.04 (0.90–1.21)	0.0	0.65	
≥10	4	0.97 (0.83–1.14)	0.0	0.69	
Adjustment for energy intake					0.43
No	2	1.07 (0.91–1.26)	0.0	0.87	
Yes	4	0.97 (0.84–1.12)	0.0	0.74	
Adjustment for BMI					0.85
No	3	1.00 (0.86–1.16)	0.0	0.38	
Yes	3	1.02 (0.87–1.20)	0.0	0.96	
Study quality^6^					0.52
Low quality	1	1.08 (0.87–1.34)	-	-	
High quality	5	0.99 (0.87–1.12)	0.0	0.82	
Prostate Cancer Mortality					
Overall	7	0.55 (0.33–0.92)	96.6	<0.001	
Subgroup analysis					
Study location					0.20
US	4	0.80 (0.70–0.92)	0.0	0.42	
Non-US countries	3	0.35 (0.14–0.89)	95.2	<0.001	
Sample size, participants					0.16
<10,000	4	0.39 (0.19–0.80)	96.3	<0.001	
≥10,000	3	0.82 (0.70–0.96)	0.0	0.70	
Adjustment for energy intake					0.33
No	5	0.45 (0.24–0.86)	93.1	<0.001	
Yes	2	0.82 (0.71–0.95)	0.0	0.53	
Adjustment for BMI					0.40
No	2	0.32 (0.05–2.07)	92.1	<0.001	
Yes	5	0.63 (0.35–1.15)	97.5	<0.001	
Study quality^6^					<0.001
Low quality	3	0.30 (0.17–0.52)	73.4	0.02	
High quality	4	0.84 (0.73–0.95)	0.0	0.83	
Prostate cancer progression					
Overall	3	0.84 (0.65–1.10)	5.4	0.35	
Localized prostate cancer					
Overall	2	0.90 (0.72–1.12)	0.0	0.53	

^1^BMI, body mass index; CI, confidence interval; RR, Relative Risk; US, United States. ^2^Number of risk estimates. ^3^Obtained from the random-effects model. ^4^Inconsistency- the percentage of variation across studies due to heterogeneity. ^5^Obtained from the Q-test. ^6^Studies with a median score of 7 or more, based on the NOS, were considered as a high-quality study.

**Figure 3:**
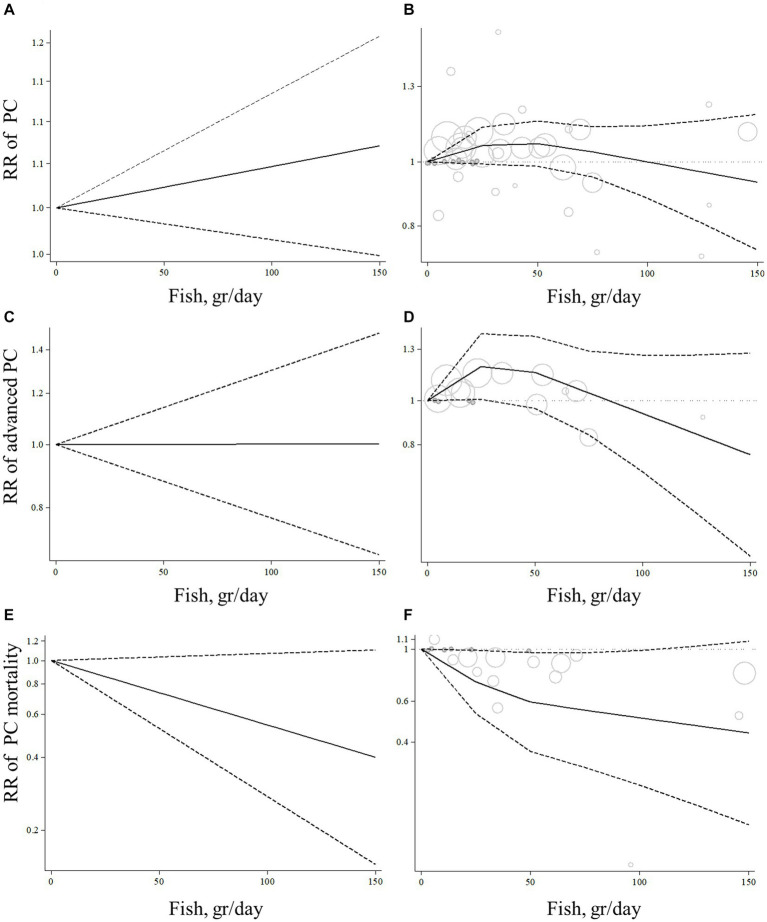
Linear and non-linear dose–response association of total fish intake (based on gr/day) with risk of prostate cancer **(A,B)**, advanced prostate cancer **(C,D)**, and prostate cancer mortality **(E,F)** in men aged ≥18  years. The solid lines indicate the spline model. The dashed lines present the 95% confidence interval; PC, prostate cancer; RR, relative risk.

Eleven articles with sufficient data were identified for inclusion in the dose–response analysis (9, 10, 13, 17–20, 23–26). The dose–response analyzes showed no significant linear (P-linearity = 0.27) and non-linear (P-nonlinearity = 0.11) association between total fish intake and the risk of prostate cancer (Figures 3A,B; Supplementary Table S3).

### Total fish intake and advanced prostate cancer

**Figure 4:**
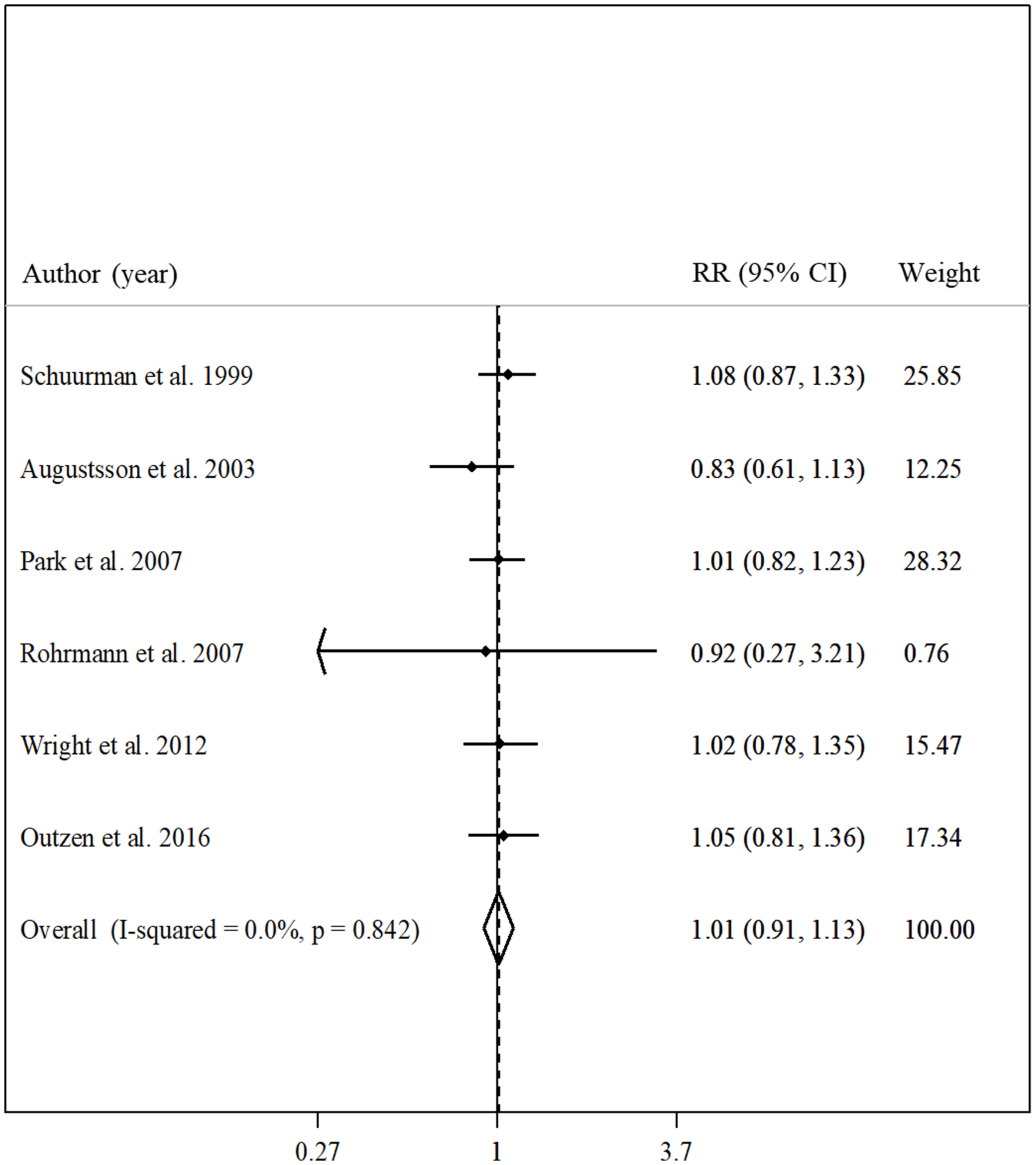
Forest plot for the association between total fish intake and risk of advanced prostate cancer in men aged ≥18 years by comparing the highest and lowest categories of fish intake. The pooled RR was obtained using a random-effects model. RR, relative risk; CI, confidence interval.

Total fish intake in relation to advanced prostate cancer was examined in 6 articles that included 190,284 participants and 3,098 cases of advanced prostate cancer (10, 19, 20, 23, 25, 32). The summary relative risk for comparing between the highest and lowest intake of total fish did not show a significant association between total fish intake and advanced prostate cancer (pooled relative risk: 1.01, 95% CI: 0.91–1.13, *p* = 0.84; Figure 4). No evidence of significant heterogeneity was found among the studies (*I*^2^ = 0.0%; *p* = 0.84). In the subgroup analyzes, heterogeneity among the studies was low among all subgroups. In addition, based on the meta-regression, we found no significant difference among the subgroups of studies in terms of the association between fish intake and the risk of advanced prostate cancer (Table 2).

Based on the dose–response analyzes on 4 articles with complete data (10, 19, 20, 23), no linear association was found between total fish consumption and risk of advanced prostate cancer (P-linearity = 0.99; Figure 3C). However, we found evidence of a non-linear association (P-nonlinearity = 0.03) so that the risk of advanced prostate cancer increased from zero to 30 gram/day fish intake, and then, we observed a risk reduction from 30 gram/day to higher amounts. It should be noted that the risk reduction of advanced prostate cancer was not statistically significant in all dosages of fish intake (Figure 3D; Supplementary Table S3).

### Total fish intake and prostate cancer mortality

**Figure 5:**
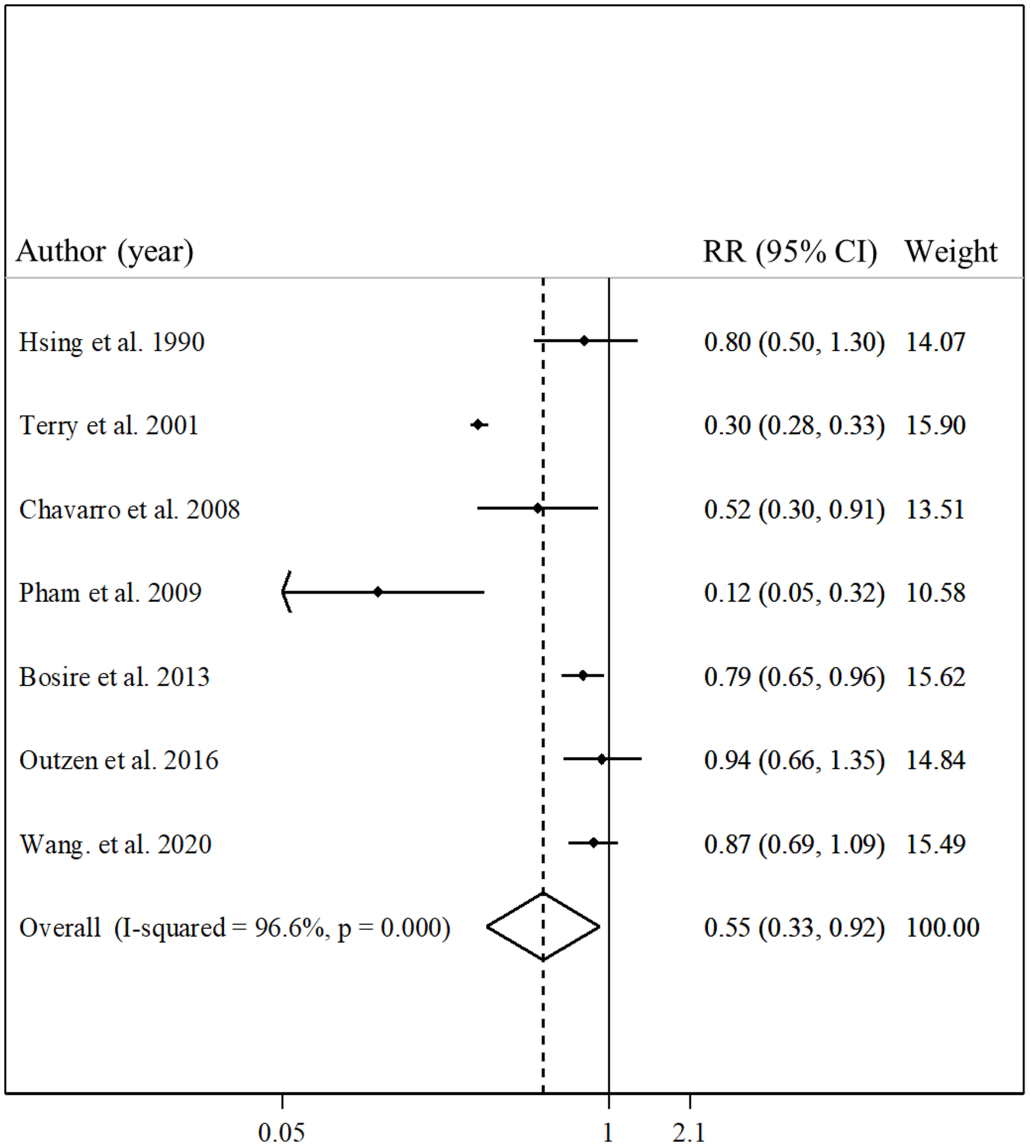
Forest plot for the association between total fish intake and risk of prostate cancer mortality in men aged ≥18 years by comparing the highest and lowest categories of fish intake. The pooled RR was obtained using a random-effects model. RR, relative risk; CI, confidence interval.

Seven articles with a total of 361,154 participants and 2,062 cases of death due to prostate cancer presented data on the association between total fish intake and risk of prostate cancer mortality (11, 13, 14, 19, 21, 27, 29). Combining relative risks of prostate cancer mortality, comparing the highest and lowest intakes of total fish, we found a significant inverse association between total fish intake and risk of prostate cancer mortality (pooled relative risk: 0.55, 95% CI: 0.33–0.92, *p* = 0.02, I^2^ = 96.6%; *p* < 0.001; Figure 5). In the subgroup analyzes, the observed heterogeneity reduced in some subgroups including studies that were conducted in the US, those that had a sample size of ≥10,000 participants, studies with high quality, and those that adjusted for total energy intake (Table 2). In addition, the meta-regression showed a significant difference between the subgroups of studies’ quality (*p* < 0.001) so the significant inverse association between fish intake and risk of prostate cancer mortality was stronger among low-quality studies compared with those with high quality (Table 2). However, the inverse association was significant in both subgroups.

Six articles had sufficient data for inclusion in the dose–response analysis (11, 13, 14, 19, 21, 29). We found a non-significant linear association between total fish intake and prostate cancer mortality (Figure 3E; P-linearity = 0.08) so that each 10 gram/day increase in total fish intake was associated with a 6% (pooled relative risk: 0.94, 95% CI: 0.87–1.00, *p* = 0.08) and each 20 gram/day increase in total fish intake was associated with a 12% (pooled relative risk: 0.88, 95% CI: 0.77–1.01, *p* = 0.08) lower risk of death due to prostate cancer. In terms of the non-linear dose–response analysis, we found no evidence of non-linearity (P-nonlinearity = 0.12; Figure 3F; Supplementary Table S3).

### Total fish intake and other outcomes

**Figure 6:**
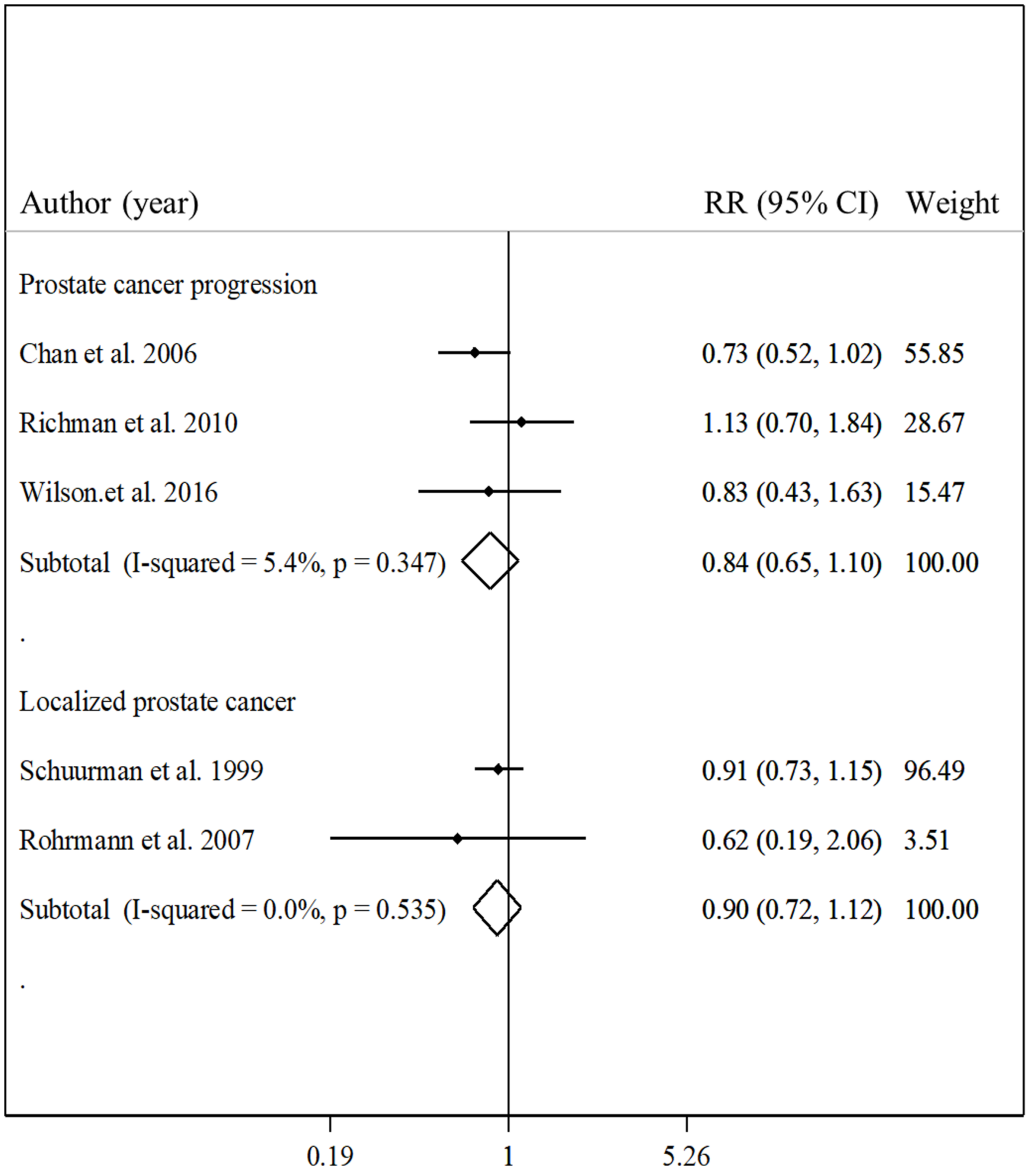
Forest plot for the associations of total fish intake with the risk of localized prostate cancer and prostate cancer progression in men aged ≥18 years by comparing the highest and lowest categories of fish intake. The pooled RR was obtained using a random-effects model. RR, relative risk; CI, confidence interval.

Three studies with a total of 3,436 participants and 613 cases of prostate cancer progression assessed the association between total fish intake and prostate cancer progression (12, 22, 31). The summary relative risk for comparing the highest with the lowest intakes of total fish showed no significant association in this regard (pooled relative risk: 0.84, 95% CI: 0.65–1.10, *p* = 0.21, I^2^ = 5.4%; *p* = 0.35; Figure 6). Regarding localized prostate cancer, we found 2 articles that included 6,059 participants and 300 cases of localized prostate cancer (23, 25). Combining relative risks from these articles, we found no significant association between total fish intake and localized prostate cancer (pooled relative risk: 0.90, 95% CI: 0.72–1.12, *p* = 0.34, I^2^ = 0.0%; *p* = 0.53; Figure 6). Since there was a limited number of studies in terms of localized prostate cancer and prostate cancer progression, performing the subgroup analyzes and dose–response analyzes was not possible.

### Sensitivity analyzes and publication bias

Sensitivity analyzes based on a random-effects model showed that excluding any single study from the analyzes did not alter the pooled relative risks. We conducted the Egger’s test for the association between total fish intake and risk of overall prostate cancer with ≥10 effect sizes and found no substantial publication bias for this association (*p* = 0.92). For other associations, due to a limited number of studies, we did not assess the publication bias.

## Discussion

In the present study, we found a significant inverse association between total fish intake and prostate cancer mortality so that each 10 or 20 gram/day increase in total fish intake was associated with a 6% or 12% lower risk of prostate cancer mortality. Regarding total, advanced, and localized prostate cancer as well as prostate cancer progression, no significant association was found with total fish intake.

Prostate cancer is one of the most common causes of death among men (1). Recently, it has been shown that dietary factors are involved in the etiology of prostate cancer (74). Among them, fish intake has received great attention because this food group contains a high amount of long-chain omega-3 fatty acids that are proposed to have anti-cancer effects (75). However, findings of cohort studies on the association between fish consumption and prostate cancer are inconclusive (9, 10, 18, 20, 27, 30). In the current meta-analysis, despite the non-significant association between total fish intake and risk of total prostate cancer, we found a significant inverse association for prostate cancer mortality. Similar to our findings, a meta-analysis conducted by Szymanski in 2010 showed that fish consumption was significantly associated with a reduced risk of prostate cancer mortality (41). Furthermore, based on the World Health Organization (WHO), the incidence of prostate cancer mortality among Mediterranean countries, where fish intake is very high, was lower than in African countries and North America (1). Also, in the National Health and Nutrition Examination Survey (NHANES), Patel et al. reported that dietary intakes of fish or omega-3 polyunsaturated fatty acids (PUFAs) were inversely associated with serum levels of prostate-specific antigen (PSA) in men (76). It is well-known that higher levels of PSA among patients with prostate cancer are associated with severe outcomes including a higher risk of mortality (77). In contrast with our findings, in a 2017 meta-analysis, Dai et al. reported that high fish consumption had no significant association with the risk of prostate cancer incidence and its mortality (40). However, some methodological problems in the Dai et al. meta-analysis make their results misleading. For instance, they combined the risk of cancer incidence with the risk of mortality. In addition, they included an ineligible study (42) which was a review of previous studies, not an original article. Moreover, Dai et al. combined the risk estimates from cohort studies with those from case–control studies that contain different biases.

In the dose–response analyzes, although we found no evidence of a significant non-linear association between fish intake and risk of prostate cancer mortality, a significant inverse association was seen in the dosages of 25 to 100 gram/day. On the other hand, we found no significant association at the dosages of >100 gram/day. However, it must be kept in mind that the estimated risk of prostate cancer mortality continued to decline after 100 gram/day, but the variability increased likely due to few studies having data at those ranges (>100 gram/day). Therefore, the lack of significant inverse association at the dosages of >100 gram/day fish intake is misleading and further studies should be done to assess the associations of these dosages with the risk of prostate cancer mortality.

In the current meta-analysis, no significant association was found between total fish intake and the risk of total and advanced prostate cancer in men. Similar to our findings, two previous meta-analyzes reported such findings for total fish intake (40, 41). In the Sung et al. study, comparing the incidence of prostate cancer between Mediterranean and non-Mediterranean countries, they found no considerable difference (1). In addition, the lack of significant association was also reported in studies investigating the effects of omega-3 supplementation. Brouwer et al. reported that alpha-linolenic acid (ALA) supplementation had no significant effect on serum PSA levels in men without prostate cancer (78). In another clinical trial, Hamazaki et al. showed that daily consumption of 2,400 milligram of eicosapentaenoic acids (EPA) for 12 weeks did not have any significant effects on serum PSA levels in healthy men (79). In another similar study, prostate cancer patients taking 2.4 gram/day of EPA did not have a different PSA recurrence rate compared to the control group (80). The lack of significant association between long-chain omega-3 fatty acids supplementation and the risk of prostate cancer was also reported in a prospective study (81).

In total, it seems that fish intake and omega-3 fatty acid intake have no significant effect on prostate cancer incidence; however, we found a protective association between fish intake and prostate cancer mortality. The observed disparity between prostate cancer incidence and its mortality might be explained by the effect of omega-3 fatty acids on the response of prostate cancer to ablation therapy. Previous studies confirmed that dietary omega-3 PUFAs increase the omega-3 content of prostate tumors (82). This effect may enhance the response of prostate cancer to ablation therapy and retard progression to androgen-independent growth (82). This mechanism was in line with findings from a trial on other sex-hormone-related cancer, in which omega-3 fatty acid supplementation (1 gram/day) improved overall survival and progression-free survival in locally advanced breast cancer patients under chemotherapy (83). Also, omega-3 PUFAs can interact with androgens, the main risk factor of prostate cancer, through their reducing effects on the levels of cholesterol. Cholesterol is the main precursor of androgens and other sex hormones (84). Omega-3 PUFAs inhibit the production of pro-inflammatory prostaglandins and hydroxyeicosatetraenoic acid (HETE) *via* the down-regulation of cyclooxygenase and lipoxygenase (33). 5-HETE stimulates proliferation and inhibits apoptosis of prostate cancer cells (85, 86). 12-HETE increases the neovascularization and proliferation of prostatic tumors (85, 86). The anti-inflammatory pathways may inhibit cell proliferation and angiogenesis in prostatic tumors and therefore reduce the cancer progression and then the risk of mortality (13, 87).

In the current meta-analysis, some included studies reported a significant inverse association between fish intake and prostate cancer (27, 30), while others found no significant association (9, 10, 13, 17–20, 23–26, 32). This inconsistency among the included studies might be explained by the different health benefits of different fishes. For instance, farmed fish contain lower levels of long-chain omega-3 fatty acids than ocean fish (88). Different types of exposures among included studies may further explain the inconsistency. In some studies, canned tuna was not included in total fish intake (23, 29), but in some others was included (10, 12, 22). However, in most studies included in the current meta-analysis, the components of total fish intake were unclear. Furthermore, different cooking and processing methods used for the preparation of fish foods may justify the inconsistency.

The current meta-analysis had some strengths. To the best of our knowledge, this was the first dose–response meta-analysis exploring the association between total fish intake with the risk of prostate cancer. Previous meta-analyzes have mainly focused on the comparison between the highest and lowest intakes of fish intake. All studies included in the current meta-analysis had a prospective design. Prospective designs can alter the possibility of recall or selection bias which could be the subject of concern in case–control studies. Also, despite the significant heterogeneity observed for some associations, we could find potential sources of heterogeneity in the subgroup analyzes. In addition, we used a random-effects model for the meta-analysis that takes the heterogeneity into account.

Despite the above-mentioned strengths, our study had some limitations that should be considered when interpreting the findings. First, although included studies controlled their analysis for several confounders, residual or unmeasured confounding factors may have affected the magnitude of the association between total fish intake and the risk of prostate cancer. Moreover, some studies did not adjust for total energy intake as a key confounding variable. However, based on the meta-regression and subgroup analyzes, we found no significant difference in our findings between the subgroups of studies with and without adjustment for energy intake. Also, the variables adjusted for prostate cancer incidence and mortality were different among the included studies. This may be a reason for the different findings obtained for incidence and mortality. Second, because of the limited number of studies, we could not assess the different types of fish including fatty fish, dark meat fish, white fish, lean fish, Shrimp, and scallops in relation to the risk of prostate cancer. Third, in the present study, a web-based search was performed in Google Scholar, but only the first 500 articles were retrieved. Therefore, missing articles in this search engine were unavoidable. Fourth, although most studies used a validated instrument for dietary assessment, five studies used a non-validated questionnaire for this purpose.

In conclusion, we found that total fish intake was associated with a reduced risk of prostate cancer mortality. Also, in the dose–response analysis, each 20 gram/day increase in total fish intake was associated with a 12% lower risk of death due to prostate cancer. Regarding the risk of total, advanced, and localized prostate cancer as well as prostate cancer progression, we found no significant association with fish intake either in the highest versus lowest comparison or in the dose–response meta-analysis. Overall, since we found no significant association between fish consumption and prostate cancer risk, we cannot recommend fish intake for prostate cancer prevention. We recommended that further studies, particularly well-designed prospective cohort studies, should assess the influence of different types of fish or sea foods on prostate cancer risk. In addition, future randomized controlled trials (RCTs) should investigate the effect of fish intake on prostate cancer outcomes in affected patients.

## Author contributions

NH-E and NE contributed to the literature search. NE and OS contributed to data extraction and data analysis and drafted the manuscript which was critically revised for important intellectual content by all authors. HA contributed to the manuscript editing and obtained funding. GA contributed to the manuscript editing. OS supervised the study. All authors have read and approved the final manuscript.

## Funding

The meta-analysis was financially supported by the Gerash University of Medical Sciences, Gerash, Iran. The funder had no role in the design and conduct of the study; collection, management, analysis, and interpretation of the data; preparation, review, or approval of the manuscript; or the decision to submit the manuscript for publication.

## Conflict of interest

The authors declare that the research was conducted in the absence of any commercial or financial relationships that could be construed as a potential conflict of interest.

## Publisher’s note

All claims expressed in this article are solely those of the authors and do not necessarily represent those of their affiliated organizations, or those of the publisher, the editors and the reviewers. Any product that may be evaluated in this article, or claim that may be made by its manufacturer, is not guaranteed or endorsed by the publisher.

## Abbreviations

ALA: alpha-linolenic acid
BMI: body mass index
CI: confidence intervals
EPA: eicosapentaenoic acids
EPIC: European Prospective Investigation into Cancer and Nutrition study
FFQ: food frequency questionnaire
HETE: hydroxyeicosatetraenoic acid
HR: hazard ratio
HPFS: Health Professionals Follow-up Study
IRR: incidence rate ratio
MESH: medical subject heading terms
MRR: mortality rate ratio
NOS: Newcastle-Ottawa scale
NIH-AARP: National Institutes of Health-American Association of Retired Persons Diet and Health Study
NLCS: Netherlands Cohort Study
NHANES: National Health and Nutrition Examination Survey
OR: odds ratio
PHS: Physician’s Health Study
PUFAs: polyunsaturated fatty acids
PSA: prostate-specific antigen
RR: risk ratio
RCTs: randomized controlled trials
US: United States
