# Supplementary material for: Fish consumption and risk of prostate cancer or its mortality: an updated systematic review and dose–response meta-analysis of prospective cohort studies
Source: Front Nutr. 2023 Aug 1;10:1221029. doi: 10.3389/fnut.2023.1221029 (PMC10427873; doi:10.3389/fnut.2023.1221029)
Supplement: Supplementary file 2 [file Data_Sheet_2.docx]

**Supplementary Table 1**: The terms used to search relevant publications on the relation between fish intake and risk of prostate cancer

| **MESH terms** | | **n** |
| --- | --- | --- |
|  | 1. “Fatty Acids, Omega-3” |  |
|  | 2. “Docosahexaenoic Acid” |  |
|  | 3. “Fishes” |  |
|  | 4. “Fish Oils” |  |
|  | 5. “Eicosapentaenoic Acid” |  |
|  | 6. “alpha-Linolenic Acid” |  |
|  | 7. “Cod Liver Oil” |  |
|  | 8. “Seafood” |  |
|  | 9. “fatty acids” |  |
|  | 10. “lipids” |  |
|  | 11. “Linoleic Acid” |  |
|  | 12. "Prostatic Neoplasms" |  |
| **Non-MESH terms** | |  |
|  | 13. “Docosahexaenoic” |  |
|  | 14. “Fish*” |  |
|  | 15. “Fish Oil” |  |
|  | 16. “Eicosapentaenoic” |  |
|  | 17. “Linolenic Acid” |  |
|  | 18. “fat” |  |
|  | 19. “Seafood” |  |
|  | 20. “fatty acid*” |  |
|  | 21. “lipid*” |  |
|  | 22. “Linoleic Acid” |  |
|  | 23. “PUFA” |  |
|  | 24. “ALA” |  |
|  | 25. “DHA” |  |
|  | 26. “EPA” |  |
|  | 27. “omega” |  |
|  | 28. “prostatic” |  |
|  | 29. “Prostate” |  |
|  | 30. “observational” |  |
|  | 31. “prospective” |  |
|  | 32. “cohort” |  |
|  | 33. “hazard” |  |
|  | 34. “longitudinal” |  |
|  | 35. “historical” |  |
|  | 36. (1 OR 2 OR 3 OR 4 OR 5 OR 6 OR 7 OR 8 OR 9 OR 10 OR 11 OR 13 OR 14 OR15 OR 16 OR 17 OR 18 OR 19 OR 20 OR 21 OR 22 OR 23 OR 24 OR 25 OR 26 OR 27) |  |
|  | 37. (12 OR 28 OR 29) |  |
|  | 38. (30 OR 31 OR 32 OR 33 OR 34 OR 35) |  |
|  | 39. (13 OR 14 OR 16 OR 17 OR 18 OR 19 OR 20 OR 22 OR 27) |  |
|  | (36 AND 37 AND 38) **In PubMed** | 1,143 |
|  | (39 AND 29 AND 38) **In Scopus** | 2,547 |
|  | (39 AND 29 AND 38) **In ISI Web of Science** | 1,131 |
|  | Duplicate papers | 708 |
| **In Google Scholar** | | 500 |
|  | “fish” and “prostate cancer”  By searching the above combination in this engine, we first sorted the results by relevance to search keywords (not time of publication) and then screened the first 500 papers to avoid missing any eligible studies. |  |
| **Total (with duplicates)** | | 5,321 |

**Supplementary Table 2**: Quality assessment of studies included in the current systematic review and meta-analysis on the association between fish intake and risk of prostate cancer^1^

|  | Representativeness of the exposed cohort | Selection of the non-exposed cohort | Ascertainment of exposure | Outcome of interest was not present at the start of the study | Energy adjustment | Controls for any additional factor | Assessment of outcome | Follow-up long enough | Adequacy of follow-up of cohorts | Total |
| --- | --- | --- | --- | --- | --- | --- | --- | --- | --- | --- |
| Allen et al. 2004 | 1 | 1 | 0 | 1 | 0 | 1 | 1 | 1 | 1 | 7 |
| Allen et al. 2008 | 1 | 1 | 0 | 1 | 1 | 1 | 1 | 1 | 1 | 8 |
| Augustsson et al.2003 | 1 | 1 | 0 | 1 | 1 | 1 | 0 | 1 | 1 | 7 |
| Bosire et al.2013 | 1 | 1 | 0 | 1 | 1 | 1 | 1 | 1 | 1 | 8 |
| Chan et al. 2006 | 0 | 1 | 0 | 1 | 1 | 1 | 1 | 1 | 1 | 7 |
| Chavarro et al. 2008 | 1 | 1 | 0 | 1 | 0 | 1 | 0 | 1 | 1 | 6 |
| Hsing et al.1990 | 1 | 1 | 0 | 1 | 0 | 1 | 1 | 1 | 1 | 7 |
| Kenfield et al. 2016 | 1 | 1 | 0 | 1 | 0 | 1 | 0 | 1 | 1 | 6 |
| Lan et al.2021 | 1 | 1 | 0 | 1 | 1 | 1 | 1 | 1 | 1 | 8 |
| Marchand et al. 1994 | 1 | 1 | 1 | 1 | 0 | 1 | 1 | 1 | 1 | 8 |
| Mills et al.1989 | 1 | 1 | 0 | 1 | 0 | 1 | 0 | 0 | 1 | 5 |
| Outzen et al. 2016 | 1 | 1 | 0 | 1 | 0 | 1 | 1 | 1 | 1 | 7 |
| Park et al.2007 | 1 | 1 | 0 | 1 | 1 | 1 | 1 | 0 | 1 | 7 |
| Pham et al.2009 | 0 | 1 | 0 | 1 | 0 | 1 | 1 | 1 | 1 | 6 |
| Richman et al.2010 | 0 | 1 | 0 | 1 | 1 | 1 | 1 | 1 | 1 | 7 |
| Rohrmann et al. 2007 | 0 | 1 | 0 | 1 | 1 | 1 | 1 | 1 | 1 | 7 |
| Sato et al. 2008 | 1 | 1 | 0 | 1 | 1 | 1 | 1 | 0 | 1 | 7 |
| Schuurman et al. 1999 | 0 | 1 | 0 | 1 | 0 | 1 | 1 | 0 | 1 | 5 |
| Severson et al.1989 | 0 | 1 | 1 | 1 | 0 | 1 | 1 | 1 | 1 | 7 |
| Terry et al. 2001 | 0 | 1 | 0 | 1 | 0 | 1 | 1 | 1 | 1 | 6 |
| Torfadottir et al. 2013 | 0 | 1 | 0 | 1 | 0 | 1 | 1 | 0 | 1 | 5 |
| Wang et al. 2020 | 0 | 1 | 0 | 1 | 1 | 1 | 1 | 1 | 1 | 7 |
| Watling et al.2022 | 1 | 1 | 0 | 1 | 0 | 1 | 1 | 1 | 1 | 7 |
| Wilson et al. 2016 | 0 | 1 | 0 | 1 | 1 | 1 | 1 | 0 | 1 | 6 |
| Wright et al.2012 | 1 | 1 | 0 | 1 | 1 | 1 | 1 | 1 | 1 | 8 |

^1^Quality assessment was done based on the Newcastle-Ottawa scale (NOS)

**Supplementary Table 3**: Relative risks and 95% CIs from the nonlinear dose-response analysis

| Fish intake (g/d) | Total PC | Advanced PC | PC mortality |
| --- | --- | --- | --- |
|  |  |  |  |
| 0 | 1 | 1 | 1 |
| 25 | 1.06 (0.99-1.13) | 1.19 (1.01-1.40) | 0.72 (0.53-0.99) |
| 50 | 1.07 (0.98-1.15) | 1.15 (0.96-1.39) | 0.59 (0.37-0.97) |
| 75 | 1.04 (0.95-1.13) | 1.04 (0.84-1.29) | 0.55 (0.31-0.96) |
| 100 | 1.00 (0.88-1.13) | 0.94 (0.70-1.26) | 0.51 (0.26-0.99) |
| 125 | 0.97 (0.81-1.15) | 0.84 (0.57-1.26) | 0.47 (0.22-1.03) |
| 150 | 0.93 (0.74-1.18) | 0.76 (0.45-1.27) | 0.44 (0.18-1.08) |
| P_nonlinearity_ | 0.11 | 0.03 | 0.12 |

Abbreviations: PC: prostate cancer- CI: Confidence Interval- g/d: gram(s) per day.
